# Three-dimensional analysis of changes in airway space after bimaxillary orthognathic surgery with maxillomandibular setback and their association with obstructive sleep apnea

**DOI:** 10.1186/s40902-018-0171-3

**Published:** 2018-11-09

**Authors:** Seung-Il Jang, Jaemyung Ahn, Jun Young Paeng, Jongrak Hong

**Affiliations:** Department of Oral and Maxillofacial Surgery, Samsung Medical Center, Sungkyunkwan University School of Medicine, 81 Irwon-ro, Gangnam-Gu, Seoul, 06351 Republic of Korea

**Keywords:** Bimaxillary orthognathic surgery, Maxillomandibular setback, Maxillary setback, Mandibular setback, Airway space, Apnea-Hypopnea Index

## Abstract

**Background:**

Bimaxillary orthognathic surgery with maxillomandibular setback is often accompanied by changes in airway space. We analyzed the changes in airway space before and after surgery and assessed their association with obstructive sleep apnea.

**Methods:**

This study is based on the cohort of 13 adult patients (9 males, 4 females, average age 23.85 years) who underwent bimaxillary orthognathic surgery with maxillomandibular setback.

We performed computed tomography and portable polysomnography before and after the surgery to assess changes in airway space and Apnea-Hypopnea Index (AHI) values (total, supine, non-supine).

**Results:**

The oropharyngeal airway volume decreased by 29% after the surgery, which was statistically significant (*p* < .05). The upper airway volume and hypopharyngeal airway volume were decreased, but not significantly (4 and 19%, respectively). The changes in airway surface area were statistically significant at all levels examined (*p* < .05). Changes in the maximum anteroposterior width of the airway were also significant at all levels (*p* < .05). However, the changes in maximum lateral width were only statistically significant at C2 level (*p* < .05). AHI values were increased after the surgery but not significantly at any position.

**Conclusions:**

Although bimaxillary surgery with maxillomandibular setback significantly reduces the airway space, it does not affect AHI values or induce obstructive sleep apnea.

## Background

Bimaxillary orthognathic surgery is a widely used treatment for skeletal malocclusion [1–3]. In addition to its positive effects for treatment of malocclusion, it also changes the anatomical locations of the soft tissues, including the muscles around the jaw and tongue base; previous studies have reported that mandibular setback surgery results in anteroposterior narrowing of the airway space, after which some patients develop the symptom of snoring [4, 5]. Recent systemic review has concluded that there is no clear association between mandibular setback surgery and occurrence of obstructive sleep apnea (OSA) [6]. Other studies have shown that bimaxillary orthognathic surgery is less likely to induce airway obstruction or OSA compared to mandibular setback surgery as a standalone procedure [7–10]. However, multilevel compensative change of airway space related following bimaxillary orthognathic surgery with maxillomandibular setback is unclear.

The purpose of this study was to assess the association between changes in the airway space after the surgery and subsequent OSA in patients who underwent bimaxillary orthognathic surgery with maxillomandibular setback. We performed a three-dimensional analysis using computed tomography to improve on the traditional airway space parameters measured using a two-dimensional cephalometric analysis. In addition, we measured AHI values before and post-surgery through polysomnography, to assess the relationship between the changes in airway space and AHI values.

## Methods

### Subjects

This study was based on 13 adult (≥ 18 years old) patients diagnosed with class III malocclusion and mandibular prognathism and treated with bimaxillary orthognathic surgery with maxillomandibular setback at the Department of Oral-Maxillofacial Surgery at Samsung Medical Center, between July 2016 and October 2017.

We did not consider the sex of the patient when determining inclusion or exclusion criteria, but excluded patients with underlying conditions such as systemic wasting disease, respiratory disease, or significant abnormalities in the airway space. And we calculated the BMI of the patients before the surgery and excluded patients with BMI values ≥ 30 kg/m^2^, as well as patients with OSA (AHI > 15 or AHI > 5 with symptoms) confirmed with polysomnography and Epworth Sleepiness Scale Questionnaire before the surgery. The study design was approved by the ethics review board (SMC 2018-06-04).

### Follow-up

Follow-up time points were set before the surgery (T0) and 7 months after the surgery (T1). All patients were imaged using facial bone computed tomography (GE LightSpeed VCT XT; General Electronics Medical Systems, Milwaukee, WI, USA) on T0 and T1. All patients were admitted the day before the surgery and underwent polysomnography with a portable polysomnograph (Stardust II; Koninklijke Philips Electronics N.V. of the Netherlands). At T1, the patients were provided with the polysomnography equipment and performed polysomnography again at home.

### Surgery procedures

Intermediate wafer was produced by model surgery, and a Le Fort I osteotomy was performed. The maxilla was repositioned according to the surgical plan, using the intermediate wafer, and was fixed semi-rigidly using a plate and screws. We then performed bilateral sagittal split ramus osteotomy and repositioned the distal segment to the pre-fixed maxilla, using the final wafer. There were no serious complications for the patients either before or after the surgery.

### Radiographic evaluation

#### Computed tomography

**Fig. 1:**
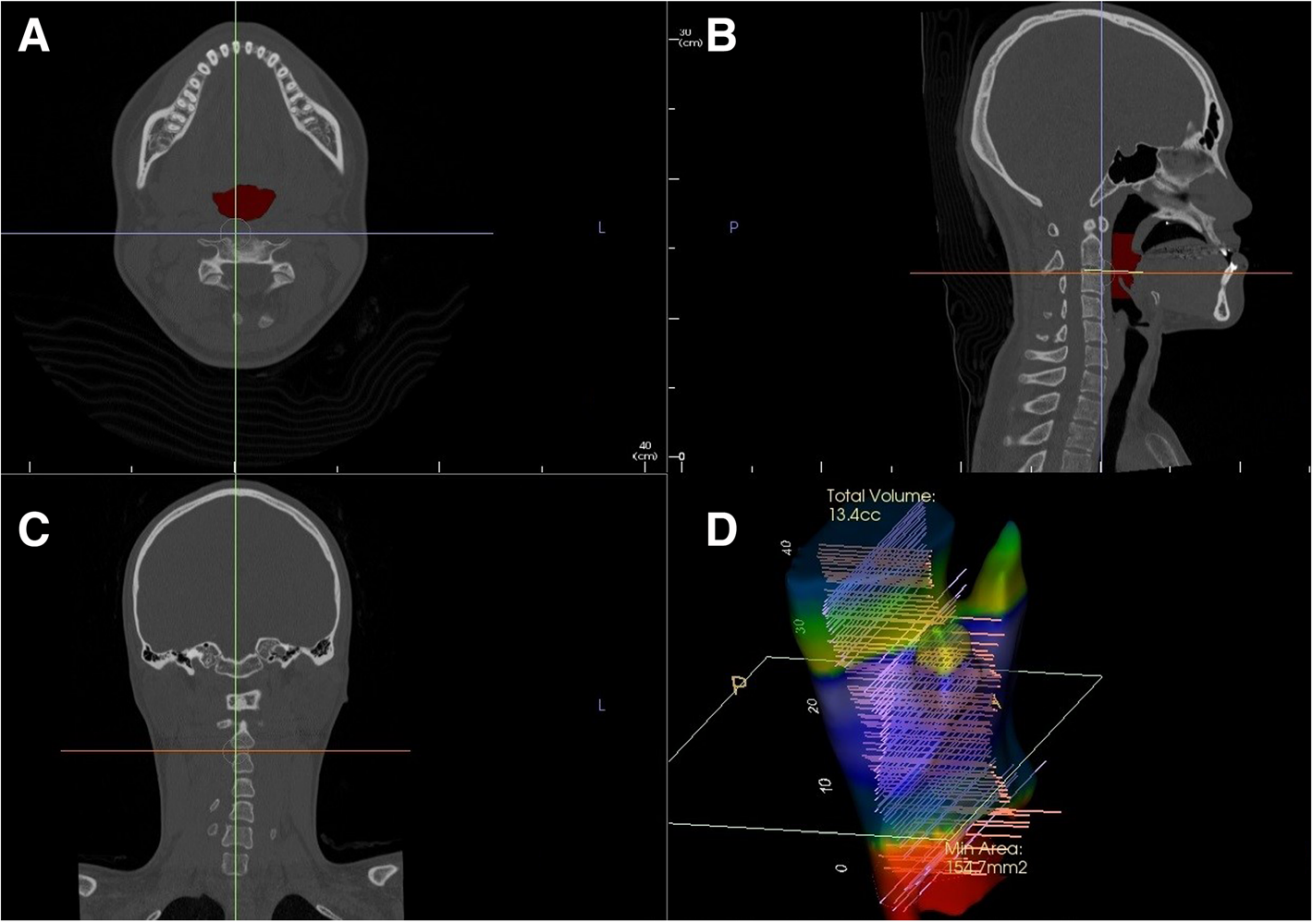
DICOM file is reconstructed into four basic views. **a** Horizontal view: In this plane, the area, maximum lateral width and maximum anteroposterior width of the airway parameters were measured. **b** Sagittal view (lateral skull view). **c** Coronal view. **d** 3D Reconstruction view: The three-dimensional form and volume of airway space are shown

The patients were imaged using panoramic radiography, cephalometric radiography (posteroanterior skull projection and lateral skull projection), and facial bone computed tomography (GE LightSpeed VCT XT; General Electronics Medical Systems, Milwaukee, WI, USA), on T0 and T1. We assessed the changes in the jawbone and airway space resulting from surgery. All radiographs were taken after ensuring centric occlusion of the teeth and removal of lower lip tension. We educated the patients on the correct postures needed for radiography, and the patients were in a supine position for facial bone computed tomography. The computed tomography data were saved in Digital Imaging and Communication in Medicine (DICOM) form, reconstituted using Invivo 5 (Anatomage Inc., San Jose, CA, USA), and analyzed in coronal, sagittal, horizontal view, and 3D reconstruction views. Using the three-dimensional reconstruction view, as reconstituted by the patient orientation function of Invivo 5, we set the Frankfort horizontal plane (FH plane, created from the orbital of both sides and right porion) and midsagittal plane (a plane that is perpendicular to the FH plane and passes through the nasion and A point) and set the head posture to be parallel with the FH plane, using the reoriented midsagittal plane as the standard. A two-dimensional image (cross section at the midsagittal plane) was used as the lateral skull view (Lat View) (Fig. 1).

#### Landmarks and measurement

**Fig. 2:**
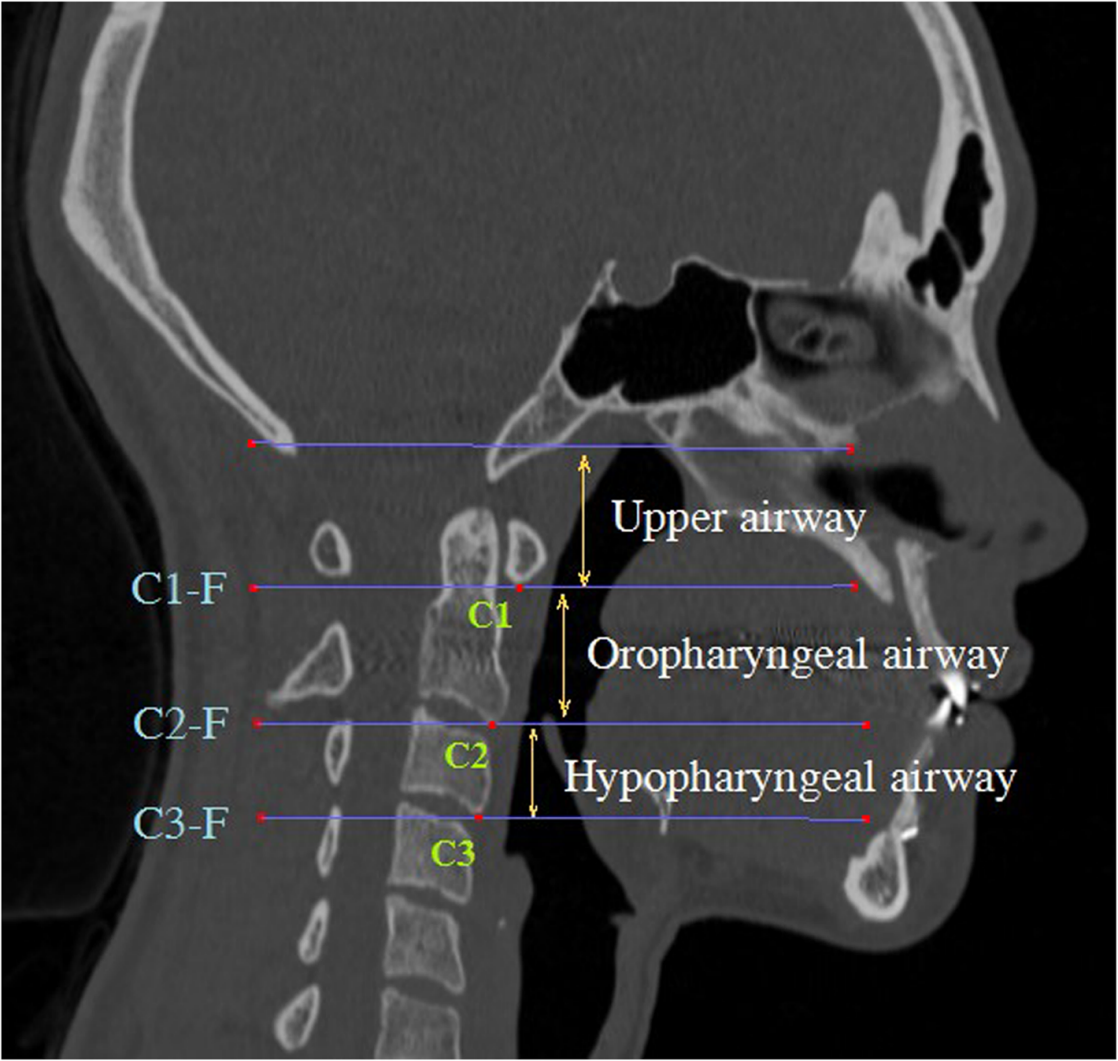
Landmarks and reference plane of total airway space. C1, C2, and C3: The lower most protruding parts of cervical vertebrae 1, 2, and 3. C1-F, C2-F, and C3-F: Planes parallel to FH plane and through C1, C2, and C3. Oropharyngeal airway: The airway space between C1-F and C2-F. Hypopharyngeal airway: The airway space between C2-F and C3-F

We defined landmarks using the Lat View. The lower most protruding parts of cervical vertebrae 1, 2, and 3 (as shown in the Lat View) were defined as the standard points C1, C2, and C3. The planes parallel to the FH plane and crossing C1, C2, and C3 were defined as the C1-FH plane (C1-F), C2-FH plane (C2-F), and C3-FH plane (C3-F), respectively. The airway space above the C1-F plane is defined as the upper airway, and the airway space between C1-F plane and C3-F plane is defined as the lower airway. The upper airway and the lower airway are combined to define the total airway. The airway space between C1-F and C2-F was defined as the oropharyngeal airway, and the airway space between C2-F and C3-F was defined as the hypopharyngeal airway (Fig. 2).

**Fig. 3:**
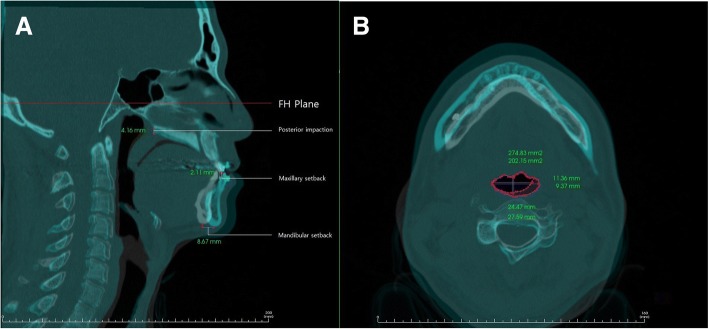
**a**. Measurements of preoperative and postoperative airway parameter changes using Invivo 5 superimposition module. Posterior impaction: The perpendicular distance to the FH plane between the posterior nasal spines (PNS) before and after surgery. Maxillary setback: The horizontal distance between the incisor tips as in parallel with the FH plane. Mandibular setback: The horizontal distance between mentons as in parallel with the FH plane. The maximum anteroposterior width (C1AP, C2AP, C3AP), **b**. maximum lateral width (C1LW, C2LW, C3LW), and the area of the cross section (C1A, C2A, C3A) on the horizontal view at C1, C2, and C3 level were measured, respectively

If an assessment of changes at an identical position from before to after the surgery (that is, from T0 to T1) was required, we used the superimposition module of Invivo 5, overlaying the CT scans from T0 and T1 on the same location and plane of the skull base layer. The airway measurement function of Invivo 5 allowed measurements of total airway volume, upper airway volume, and lower airway volume (oropharyngeal airway volume, hypopharyngeal airway volume) before and after the surgery (Fig. 1). We measured the maximum anteroposterior width (C1AP, C2AP, C3AP), maximum lateral width (C1LW, C2LW, C3LW), and the corresponding cross-sectional areas (C1A, C2A, C3A) at the cross section where the C1-F, C2-F, and C3-F planes passed through the airway space, for T0 and T1. Using a Lat View of the overlaid CT scans, we defined the perpendicular distance to the FH plane between the posterior nasal spines (PNS) before and after the surgery as the amount of posterior impaction. The distance between the incisor tips as in parallel with the FH plane was defined as the maxillary setback. In the same way, the distance between the mentons was defined as the mandibular setback (Fig. 3).

### Polysomnography evaluation

All patients underwent polysomnography after being admitted on the day before the surgery. The patients completed a survey for polysomnography before the test. Height and weight information obtained from the survey were used to calculate the BMI of the patients.

After the surgery, the patients took the portable polysomnography equipment (Stardust II; Koninklijke Philips Electronics N.V. of the Netherland) home and performed the test again on T1.

**Fig. 4:**
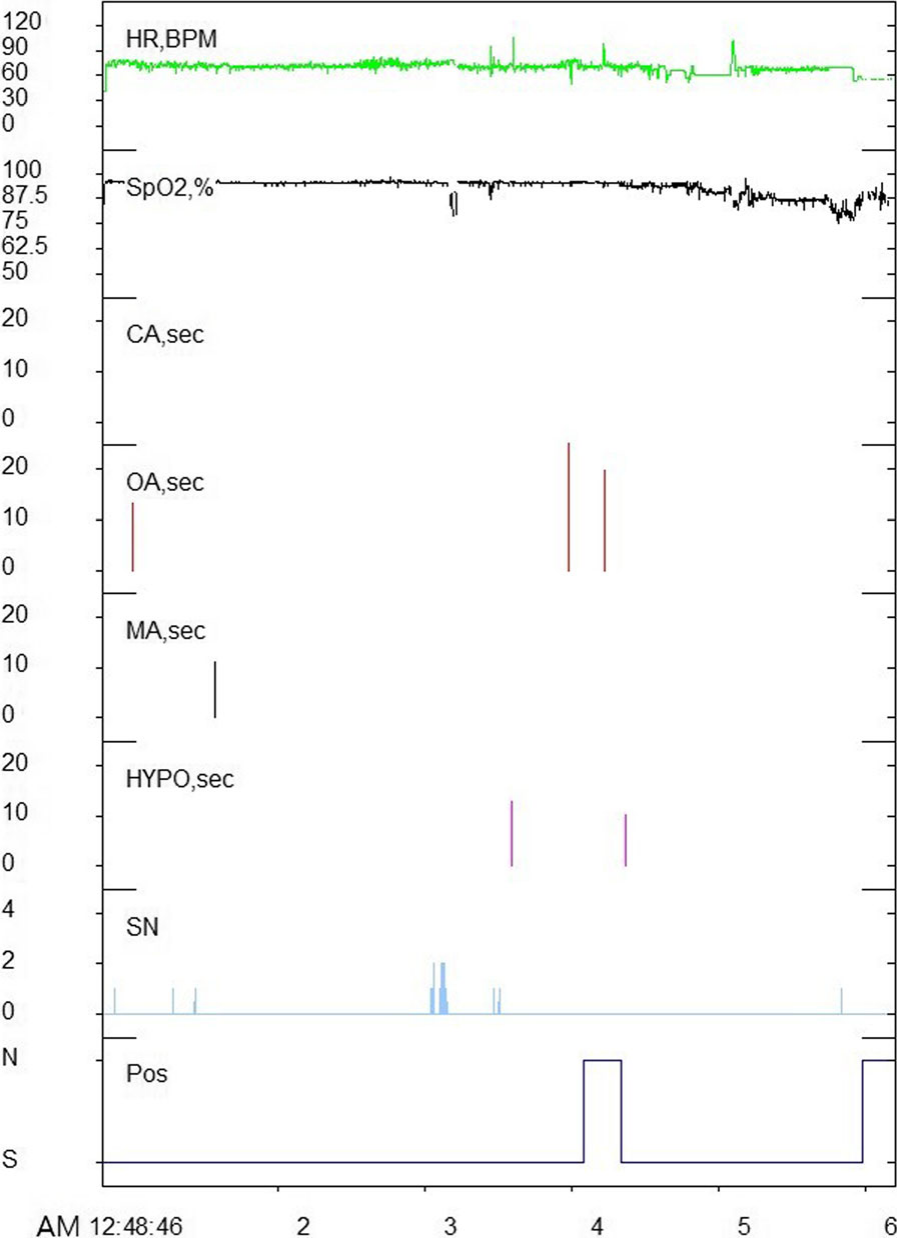
Analysis of polysomnography. While the patient was sleeping, heart rate, beats per minute, SpO_2_, central apnea, obstructive apnea, mixed apnea, hypopnea, snoring, and sleep posture were recorded

Information on heart rate, beats per minute, SpO_2_, central apnea, obstructive apnea, mixed apnea, hypopnea, snoring, and sleep posture was recorded during the test. Data from the equipment were validated and modified (if needed) by a single technologist. AHI values (total, supine, non-supine) and lowest SpO_2_ were used in this study (Fig. 4).

### Variables and statistical analysis

We performed statistical analyses of each parameter before and after the surgery using IBM SPSS Statistics (version 23.0). Changes in the parameters of airway space (total, upper, lower, oropharyngeal, and hypopharyngeal airway volumes, C1AP, C2AP, C3AP, C1LW, C2LW, C3LW, C1A, C2A, C3A) and the polysomnography parameters (total, supine, non-supine AHI, lowest SpO_2_) before and after the surgery were assessed for statistical significance using the paired *t* test or Wilcoxson’s signed rank test (with significance level *α* = 0.05). Furthermore, the relationship between the airway space parameters (oropharyngeal, hypopharyngeal and upper airway volumes, C1AP, C2AP, C3AP, C1LW, C2LW, C3LW, C1A, C2A, C3A) and the amount of mandibular setback, maxillary setback, and posterior impaction was assessed via Spearman’s correlation analysis.

## Results and discussion

The average age of the patients in this study was 23.92 years; of the 13 patients, 9 patients were males and 4 were females. The average pre-surgery BMI was 24.86 ± 2.48 kg/m^2^ (Table 1), and none of the patients exhibited underlying OSA prior to the surgery (12 patients had AHI ≤ 5 and 1 patient had an AHI of 5.1; none of the patients had systemic disorders or daytime sleepiness). The average post-surgery (T1) BMI was 24.54 ± 2.19 kg/m^2^, and the difference of the BMI was not statistically significant (Table 1).

**Table 1:** General characteristics of subjects

Number of Subjects (Male/Female)	13 (9/4)	
Age (Years)	23.92 ± 5.15 (21 – 38)	
BMI (Kg/m^2^)		
(T0)	24.86 ± 2.45 (21.61 – 29.86)	*p*> 0.05 (Paired t-test)
(T1)	24.54 ± 2.19 (22.04 – 28.74)	
Maxillary setback(mm)		1.53 ± 1.05 (0 – 3.11)
Posterior Impaction(mm)		3.91 ± 1.68 (0 – 6.68)
Mandibular setback(mm)		10.15 ± 3.27 (3.85 – 15.79)

*T0* before surgery, *T1* 7 months after surgery

Data are shown as mean ± SD (range)

The average amount of mandibular setback at the time of the surgery was 10.15 ± 3.27 mm, and the average amount of maxillary posterior impaction was 3.91 ± 1.68 mm from the posterior nasal spine (PNS). The average amount of maxillary setback was 1.53 ± 1.05 mm toward the posterior end (Table 1).

### Airway parameters

The average values of the airway space parameters (C1AP, C2AP, C3AP, C1LW, C2LW, C3LW, C1A, C2A, and C3A; the upper airway volume; lower airway volume; and oropharyngeal airway volume; and hypopharyngeal airway volume) measured before and after the surgery (on T0 and T1) are shown in Table 2.

**Table 2:** Morphologic change of the airway space

	T0 Mean	T1 Mean
Length (mm)		
C1AP*	13.15 ± 4.56	10.11 ± 3.69
C2AP*	13.95 ± 4.36	10.99 ± 3.85
C3AP*	14.63 ± 3.50	12.29 ± 4.92
C1LW	23.28 ± 4.63	20.38 ± 4.38
C2LW*	27.52 ± 6.70	24.13 ± 4.31
C3LW	31.86 ± 7.44	27.76 ± 8.50
Area (mm^2^)		
C1A*	237.79 ± 79.37	167.68 ± 67.05
C2A*	290.04 ± 128.14	209.87 ± 84.74
C3A*	283.51 ± 80.07	219.92 ± 84.11
Volume (mm^3^)		
Upper airway	6.95 ± 2.80	6.70 ± 2.27
Lower airway*	9.01 ± 2.71	6.78 ± 1.87
Oropharyngeal airway*	4.87 ± 1.80	3.48 ± 0.92
Hypopharyngeal airway	3.98 ± 1.34	3.21 ± 1.28
Total airway	15.95 ± 4.82	13.48 ± 3.54

*T0* before surgery, *T1* 7 months after surgery

*p < 0.05 (paired t-test, Wilcoxon`s signed rank test)

Data are shown as mean ± SD (range).

*The oropharyngeal airway volume was reduced by 29% after the surgery, which was statistically significant. The upper airway volume was reduced by 4%, and hypopharyngeal airway volume was reduced by 19% but not significantly*.

We compared the maximum anteroposterior length, lateral width, and the area of the cross section of the airway space at the C1, C2, and C3 levels before and after the surgery (T0 and T1). At the C1 and C2 level, the maximum anteroposterior width (C1AP and C2AP) and the surface area (C1A and C2A), the maximum lateral width of C2(C2LW) showed statistically significant reductions. However, the C1LW showed not statistically significant reduction. At the C3 Level, C3AP and C3A showed statistically significant reductions. However, the difference of the C3LW was not statistically significant.

### Polysomnography

The average values of the polysomnographic data are shown in (Table 3). The average values of the AHI (total) and AHI (supine) increased by 2.51 and 2.92, respectively, after the surgery. The average value of the AHI (non-supine) decreased by 0.54 after the surgery. But all these values were not statistically significant. The average values of the lowest SpO_2_ changed a little after the surgery. Details of the data for each patient (on T0 and T1) are shown in Table 5

**Table 3:** the relationship between the airway space parameters and the amount of jaw movement

	Mandibular setback	Maxillary setback	Posterior impaction
Volume (mm^3^)			
Oropharyngeal airway	0.2466	0.7602	0.2079
Hypopharyngeal airway	0.7074	0.9571	0.7339
Upper airway	0.6286	0.2058	0.6672
Length (mm)			
C1LW	0.578	0.9002	0.2749
C1AP	0.9432	0.5267	0.014*
C2LW	0.8028	0.3316	0.1167
C2AP	0.344	0.0531	0.7137
C3LW	0.3943	0.3316	0.8865
C3AP	0.2547	0.2601	0.687
Area (mm^2^)			
C1A	0.9716	0.7878	0.1142
C2A	0.3637	0.6011	0.7818
C3A	0.9716	0.4568	0.9289

*p < 0.05 (Spearman`s correlation analysis)

.

## Discussion

Maxillomandibular setback causes narrowing of the airway space**.** On the contrary, maxillomandibular advancement returns the narrowed airway structure to a normal state, which consequently relieves the symptoms of OSA. Vigneron et al. [11] performed maxillomandibular advancement surgery (MMA) on OSA patients and obtained a 50–80% reduction in AHI values after the surgery. Another study by Varghese et al. [12] showed an 83% reduction in AHI value 6.7 months after the surgery. These results suggest that MMA can significantly reduce the AHI value, and therefore, it may be considered that maxillomandibular setback surgery could significantly increase the AHI value.

We performed a three-dimensional analysis of the changes in airway space after the maxillomandibular setback surgery, and all patients underwent polysomnography, to assess not only the changes in airway space but also the association between the changes in airway space and the AHI value. The results of this study demonstrated a decrease in all airway space parameters after the surgery (Table 2). However, no significant increase was observed in AHI values in all positions (total, supine, non-supine) (Table 4).

**Table 4:** Polysomnographic data

	T0	T1	
AHI (total)	2.24 ± 1.24	4.75 ± 5.91	*p*> 0.05 (Paired t-test)
AHI (supine)	2.45 ± 1.48	5.37 ± 6.31	
AHI (non-supine)	1.55 ± 1.81	1.01 ± 1.53	
Lowest oxygen saturation	93.92 ± 2.60	93.38 ± 5.36	

*T0* before surgery, *T1* 7 months after surgery

Data are shown as mean ± SD (range)

There is an ongoing debate on the occurrence of OSA after bimaxillary orthognathic surgery [13].

In the study by Gokce et al. [4], nasopharyngeal, retropalatal dimension, and the volume of the superior pharyngeal airway space increased due to the maxillary advancement. However, the anteroposterior width and the volume of the oropharyngeal, hypopharyngeal space decreased after the bimaxillary orthognathic surgery due to the mandibular setback. Total airway volume has increased, and the AHI value decreased after the surgery. The average maxillary advancement and mandibular setback in the study by Gokce et al. [4] were 5 ± 2.2 mm and 6.5 ± 2.7 mm, respectively.

In contrast, the study by Lee et al. [14] showed a decrease in airway space and increase in AHI after bimaxillary orthognathic surgery, with the average maxillary setback and mandibular setback of 1.01 ± 1.54 mm and 8.23 ± 3.59 mm, respectively. The difference between these studies is thought to come from the difference in maxillary movement (5 ± 2.2 mm and − 1.01 mm) despite a similar mandibular setback amount.

This also suggests that maxillary advancement can improve the condition of reduced airway space caused by mandibular setback. Thus, the maxillary advancement can improve the OSA. The meta-analysis of related studies by He J et al. [9] further supports this observation. In this study, no statistically significant decrease in the upper airway volume was observed after combined therapy of mandibular setback and maxillary advancement. However, statistically significant differences in the nasopharyngeal airway volume and upper air way volume were observed when compared to mandibular setback surgery alone. However, no statistically meaningful variations existed in the volume of the oropharynx and hypopharynx compared to mandibular setback surgery alone.

Kim et al. [15] reported that the direction and amount of maxillary movement has a significant effect on the changes in airway space after the surgery. Specifically, posterior impaction and advancement of the maxilla significantly increases the retropalatal area which corresponds to the upper airway in this study, which compensates for the reduction in retropalatal area caused by mandibular setback. This could also be demonstrated in our study as the posterior impaction showed statistically significant association to the changes in the C1AP (Table 5). C1AP was an airway parameter which was found to decrease significantly after the surgery in this study.

**Table 5:** All of the data for each patient (parameters of the airway space, AHI, the amount of jaw movement)

	Volume (mm^3^)						Area (mm^2^)						Maximum Lateral width (mm)						Maximum anteroposterior width (mm)						AHI values (supine, non-supine, total) and lowest SpO2								The amount of jaw movement(mm)		
	T0			T1			T0			T1			T0			T1			T0			T1			T0				T1				Maxilla		Mandible
Pt	C1F-C2F	C2F-C3F	Upper airway	C1F-C2F	C2F-C3F	Upper airway	C1A	C2A	C3A	C1A	C2A	C3A	C1LW	C2LW	C3LW	C1LW	C2LW	C3LW	C1AP	C2AP	C3AP	C1AP	C2AP	C3AP	Supine	Non-Supine	Total	SpO2	Supine	Non-Supine	Total	SpO2	setback	Posterior impaction	setback
1	6.90	4.70	7.10	3.80	4.00	5.60	156.33	151.83	247.45	86.62	79.15	166.65	32.04	31.19	37.28	24.53	25.34	35.24	7.01	6.73	11.69	4.10	3.51	8.47	3.00	3.30	3.10	96.00	4.30	0.00	4.30	96.00	0.00	5.95	10.10
2*	3.20	2.70	6.40	3.00	3.30	6.3	227.63	288.47	273.30	168.69	281.21	233.05	13.17	25.50	29.27	22.63	25.69	28.03	23.21	12.61	13.23	10.32	12.31	12.89	1.00	2.10	1.10	93.00	21.40	2.00	19.20	94.00	1.13	5.03	10.22
3	4.30	5.30	4.30	2.00	3.10	2.50	214.69	309.62	281.18	95.71	161.31	204.32	21.43	23.18	27.82	13.01	16.78	21.72	13.39	17.19	12.25	9.93	14.25	8.15	4.10	0.00	2.70	95.00	2.10	0.00	2.10	92.00	3.11	4.17	13.01
4	3.80	3.60	3.80	3.30	3.90	6.50	137.25	315.39	264.42	175.23	242.82	225.57	19.55	24.02	30.82	25.51	28.50	32.73	7.71	16.16	15.67	7.46	11.68	13.91	1.70	0.00	1.70	96.00	1.50	0.00	1.50	96.00	1.77	2.73	15.79
5	9.00	4.30	9.30	5.00	2.90	7.20	428.24	427.62	246.06	243.56	259.18	288.74	28.56	30.46	29.44	23.72	28.72	33.47	16.66	17.85	13.80	12.38	11.42	13.32	3.60	2.00	3.20	95.00	0.30	1.30	0.50	95.00	1.56	2.73	10.31
6	3.80	5.10	7.70	4.80	5.50	6.90	200.19	272.85	249.60	184.27	254.36	245.67	24.46	26.52	12.85	23.74	27.28	12.00	9.44	15.41	23.87	9.43	13.99	24.44	2.40	2.30	2.30	96.00	3.50	3.90	3.60	96.00	2.66	0.00	3.85
7	4.40	2.40	1.20	4.10	2.50	9.00	256.32	181.78	316.14	174.61	151.93	144.56	24.90	27.38	31.19	17.53	25.15	30.12	14.08	10.09	15.05	12.75	6.84	7.60	1.80	0.00	1.80	94.00	1.90	0.00	1.80	96.00	0.00	3.49	10.61
8	4.10	2.80	7.50	3.10	5.00	10.30	204.87	302.64	165.52	121.81	295.96	278.45	22.51	19.77	31.62	18.64	17.72	34.81	12.08	18.00	9.79	6.37	16.63	12.06	1.90	2.00	1.90	89.00	6.60	0.00	1.90	93.00	1.73	6.68	9.72
9	5.80	3.30	7.80	3.50	2.50	6.00	202.05	271.50	317.39	126.27	211.80	237.57	24.46	24.92	38.74	20.92	22.24	36.51	8.69	14.96	15.16	7.13	11.37	13.60	5.70	1.40	5.10	95.00	8.20	4.20	6.80	95.00	2.11	4.22	8.79
10*	2.90	4.40	9.40	3.10	0.80	8.00	230.79	227.20	295.55	229.95	117.89	57.44	22.08	42.17	41.65	22.08	25.69	16.67	12.89	9.02	13.40	12.61	7.22	4.38	1.10	0.00	1.10	94.00	15.10	0.00	15.40	76.00	1.00	3.00	4.94
11	7.00	6.80	12.40	4.10	4.00	9.80	297.24	616.61	517.55	179.97	354.39	412.57	25.26	38.73	41.75	17.36	29.43	38.26	14.16	20.67	18.57	11.13	14.15	16.49	3.00	0.80	2.40	88.00	0.80	0.00	0.40	95.00	0.00	5.10	13.51
12	3.40	2.10	6.20	1.90	1.90	3.30	189.00	103.68	252.78	81.33	85.41	196.93	19.02	19.55	30.69	11.86	17.96	19.28	12.75	7.69	12.99	9.03	6.37	13.53	2.60	6.30	2.70	96.00	3.80	0.00	3.80	95.00	2.61	3.58	8.60
13	4.70	4.20	7.20	3.50	2.30	5.70	346.71	301.33	258.63	311.76	232.90	167.39	25.14	24.36	31.06	23.38	23.17	22.07	18.83	14.94	14.72	18.83	13.19	10.92	0.00	0.00	0.00	94.00	0.30	1.70	0.40	95.00	2.23	4.15	12.56

*T0* before surgery, *T1* 7 months after surgery, *Pt* patient

*patient who showed a remarkable increase in AHI value

In the study by Lee et al. [16], the average posterior impaction from the posterior nasal spine in the study moved upward by 5.27 ± 2.58 mm, while the average mandibular setback(from the menton) was 10.18 ± 4.50 mm. No statistically significant differences were found in the total airway volume. However, the upper airway volume has increased (12.35%). The results may suggest that posterior impaction can also improve the condition of reduced airway space caused by mandibular setback. However, the study by Lee et al. [17] that controlled the horizontal movement of the maxilla reported that there is a minimal effect of maxillary posterior impaction on the nasopharyngeal airway. In this regard, it can be hypothesized that posterior impaction has a relatively lower impact on the increase in the upper airway parameters than the horizontal movement of the maxilla.

In summary, combined therapy of mandibular setback and maxillary advancement or posterior impaction can reduce the obstruction of airway space compared to mandibular setback surgery alone. The movement of maxilla is related to the changes in the parameters of the upper air space, which shows an increasing tendency for the maxillary advancement or the posterior impaction. Mandibular setback is related to changes in the parameters of the lower air space, especially oropharyngeal air space, which shows a decreasing tendency.

A report by Jeon et al. [18] found that the narrowed pharyngeal airway after bimaxillary orthognathic surgery mostly recovered as time passed. Kitagawara et al. [19] found that patients exhibited oxygen desaturation during sleep immediately after the surgery, but the symptoms were mostly relieved within the next month. These findings suggest that the human body adapts to sudden changes in the body as time passes, allowing patients to recover.

In the study by Schendel et al. [20], total airway volume increased by 2.5 times in patients with MMA due to severe OSA. In addition, the retropalatal region increased by 3.5 times and the retroglossal region by 1.5 times. On the other hand, in our study, the total air volume decreased by about 15%. The retropalatal region decreased by 29%, and retroglossal region by about 28%. This shows that the increase in the change in airway space caused by MMA for the treatment of OSA is much greater than the decrease in the changes in airway space caused by maxillomandibular setback surgery. This also suggests that the change in air space caused by maxillomandibular setback surgery is not large enough to affect AHI value. Also, the patients treated with MMA surgery were relatively elderly patient people (mean age 48.3 ± 10.8 years) who have severe OSA and require surgical intervention due to lack of response to other therapies, while the patients in our study did not have underlying OSA before surgery and were relatively young people (mean age 23.9 ± 5.2) who sought surgical treatment for skeletal class III malocclusion. Therefore, a simple comparison should not be made between these two studies and their outcomes.

Many studies have also demonstrated that bimaxillary orthognathic surgery does not induce OSA. However, obese patients or patients with large mandibular setback have higher risk of OSA after the surgery, and therefore, surgeons should pay attention to possible changes in airway space when planning the mandibular setback surgery. In this study, two patients showed a remarkable increase in total AHI value (before the surgery, 1.0 and 1.10, no evidence of OSA; after the surgery, 19.2 and 15.40, moderate OSA) (Table 5). The two patients complained of postoperative snoring and daytime sleepiness. There was no specific reduction in the volume or sectional area in the second patient; however, the C1AP reduction was the highest among all patients treated. Patient 10 had the highest reduction change in volume, width, and area at the C2 and C3 levels among all patients. In both patients, the amounts of the maxillary setback and the mandibular setback were not particularly high. This could suggest that even if the amount of surgery is not particularly high, various factors can lead to serious decrease in the airway space, which could lead to obstructive sleep apnea. Furthermore, screening of risk factors that can induce OSA in these patients should be performed before the surgery [6, 21]. Even in this study, there was one patient who showed a remarkable increase in total AHI value (before the surgery 1.0, no evidence of OSA; after the surgery, 19.2, severe OSA).

## Conclusions

In this study, we selected a cohort of patients who underwent bimaxillary orthognathic surgery with maxillomandibular setback to assess the association between changes in airway space after the surgery and the occurrence of OSA, using facial bone computed tomography and portable polysomnography. We report that although bimaxillary surgery with maxillomandibular setback induces a significant reduction of the airway space, it does not affect AHI values or induce OSA.

## Abbreviations

AHI: Apnea-Hypopnea Index
C1A, C2A, C3A: Cross-sectional areas
C1AP, C2AP, C3AP: Anteroposterior width
C1LW, C2LW, C3LW: Maximum lateral width
Lat View: Lateral skull view
MMA: Maxillomandibular advancement surgery
PNS: Posterior nasal spine
